# A left lung abscess with a displaced subsegmental bronchus and anomalous pulmonary artery and vein: a case report

**DOI:** 10.1186/s40792-019-0627-4

**Published:** 2019-04-23

**Authors:** Kazuto Ohtaka, Nozomu Iwashiro, Kazunori Watanabe, Tomoko Mizota, Ryo Takahashi, Masato Suzuoki, Kazuteru Komuro, Masanori Ohara, Kichizo Kaga, Yoshiro Matsui

**Affiliations:** 1 Department of Surgery, National Hospital Organization Hakodate National Hospital, 18-16, Kawahara-cho, Hakodate, Hokkaido 041-8512 Japan; 2 Department of Cardiovascular and Thoracic Surgery, Hokkaido University Graduate School of Medicine, Sapporo, Hokkaido Japan

**Keywords:** Displaced bronchus, Eparterial bronchus, Pulmonary artery, Pulmonary vein, Accessory fissure, Anomaly, Variation, Preoperative diagnosis, Preoperative identification

## Abstract

**Background:**

Since a displaced bronchus related to the left upper lobe is an uncommon anatomical anomaly, it has a risk of being accidentally resected during left upper lobe resection unless they are identified preoperatively. A case of video-assisted thoracic surgery (VATS) segmentectomy that was safely performed under preoperative identification of a displaced subsegmental bronchus and anomalous pulmonary vessels is presented.

**Case presentation:**

A 48-year-old woman visited our hospital because of an abnormal shadow on a radiograph on a health check. The chest computed tomography (CT) showed a multicystic mass with a diameter of 35 mm on dorsal interlobar parenchyma between the S^1+2^ and S^6^ segments in the left lung. The three-dimensional (3D) CT with multiplanar reconstruction showed that B^1+2^b+c passed to the dorsal side of the left main pulmonary artery (PA), which was considered a displaced bronchus. The branch of A^6^ arose from the left main PA at the level of the branches of A^3^ and A^1+2^, more proximal than the normal anatomy, and passed to the dorsal side of a displaced B^1+2^b+c. The branch of V^1+2^ passed between B^6^ and the bronchus to the basal segment and joined V^6^ at the dorsal side of the pulmonary hilum. Intraoperative findings of the anatomy of the bronchi and pulmonary vessels were exactly the same as the preoperative 3D CT findings, so segmentectomy of S^1+2^b+c and S^6^ by VATS was performed safely. Then there were accessory fissures between S^1+2^ and S^3^ and between S^6^ and the basal segment. The pathological diagnosis was a left lung abscess.

**Conclusions:**

A preoperative 3D CT may be helpful for identifying anatomical anomalies. An anatomical anomaly should be suspected if accessory fissure is found during surgery.

## Background

Abnormalities of the bronchi have been classified into supernumerary bronchi, displaced bronchi, and congenital cystic diseases [1]. The prevalence of these abnormalities is 0.64% [2]. In abnormalities of the bronchi, those related to the right upper lobe accounted for 75%, and those related to the left upper lobe were rare [2]. The prevalence of a displaced bronchus to the left upper division demonstrated by bronchography is 0.3 to 0.5% [3, 4]. Thus, a displaced subsegmental bronchus to the left upper division appears to be less common [5]. The most common displaced bronchus to the left upper division is that arises from the left main bronchus (LMB) and passes to the dorsal side of the left main pulmonary artery (PA) [6]. Therefore, unless this anatomical anomaly is identified preoperatively, a displaced bronchus can sometimes be accidentally resected while dividing the left upper and lower lobes [7, 8]. A case in which video-assisted thoracic surgery (VATS) segmentectomy was safely performed under preoperative identification of a displaced subsegmental bronchus and anomalous PA and pulmonary vein (PV) is presented.

## Case presentation

**Fig. 1:**
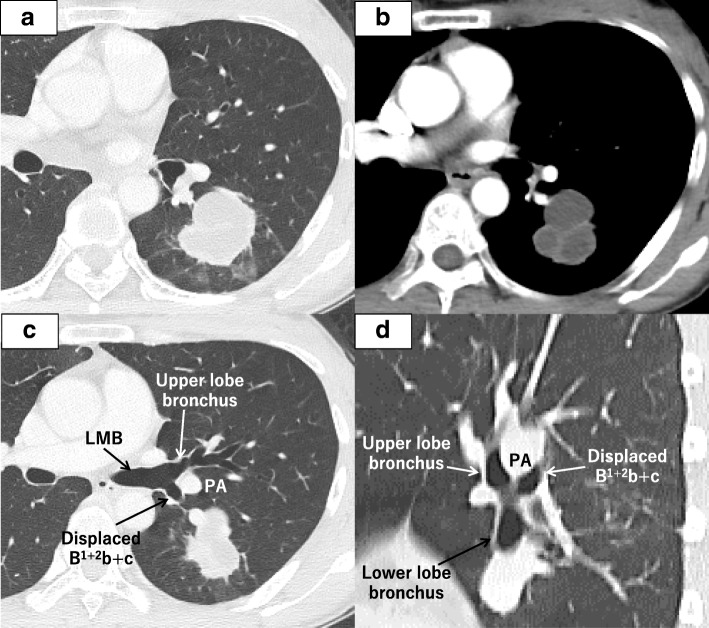
Chest computed tomography. There is a multicystic mass with a diameter of 35 mm on the dorsal interlobar parenchyma between the S^1+2^ and S^6^ segments in the left lung (**a**, **b**). The displaced bronchus arises from the left main bronchus and passes to the dorsal side of the left main pulmonary artery (**c**, **d**). LMB, left main bronchus; PA, pulmonary artery

**Fig. 2:**
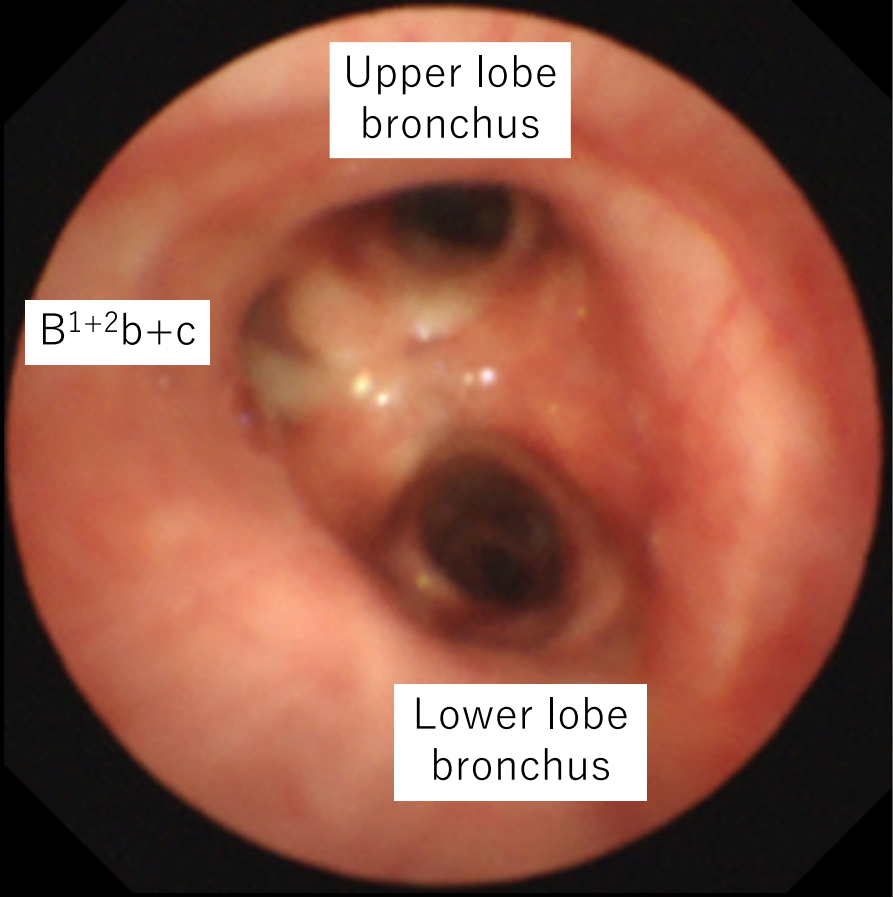
Bronchoscopic imaging. There are three bronchi branched from the left main bronchus, a branch of the lower lobe and two branches of the upper lobe

**Fig. 3:**
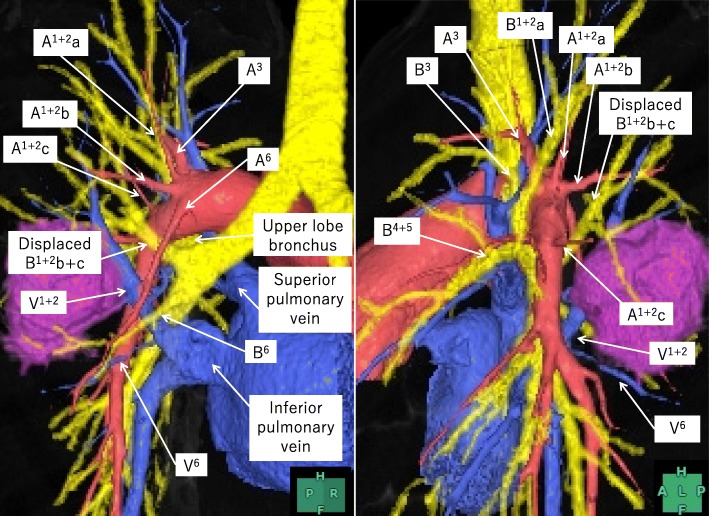
Three-dimensional computed tomography with multiplanar reconstruction. The displaced B^1+2^b+c passes to the dorsal side of the left main pulmonary artery. The branch of A^6^ arises from the left main pulmonary artery at the level of the branches of A^3^ and A^1+2^ and passes to the dorsal side of the displaced B^1+2^b+c. The branch of V^1+2^ passes between B^6^ and the bronchus to the basal segment and joins V^6^ at the dorsal side of the pulmonary hilum

A 48-year-old woman with no smoking history visited another hospital twice because of cough, 5 and 9 years earlier. The chest X-ray and computed tomography (CT) showed a nodule with a diameter of about 20 mm in the left lung that was suspected to be a bronchial cyst. She had not since visited the hospital. She finally came to our hospital because of an abnormal shadow on a radiograph on a health check. The chest CT (Revolution EVO; GE Healthcare, Tokyo, Japan) showed a multicystic mass without irregular wall thickness and a diameter of 35 mm on the dorsal interlobar parenchyma between the S^1+2^ and S^6^ segments in the left lung (Fig. 1). The bronchoscopy showed that three bronchi branched from the LMB, a branch of the lower lobe and two branches of the upper lobe (Fig. 2). No histological diagnosis was obtained by bronchoscopic biopsy. The three-dimensional (3D) CT with multiplanar reconstruction by a standalone workstation (SYNAPSE VINCENT; Fujifilm, Tokyo, Japan) showed that B^1+2^b+c passed to the dorsal side of the left main PA, which was considered a displaced bronchus (Fig. 3). The branch of A^6^ arose from the left main PA at the level of the branches of A^3^ and A^1+2^, more proximal than the normal anatomy, and passed to the dorsal side of the displaced B^1+2^b+c. The branch of V^1+2^ passed between B^6^ and the bronchus to the basal segment and joined V^6^ at the dorsal side of the pulmonary hilum. Although the preoperative diagnosis predicted benign disease, a bronchial cyst, surgical resection was performed for the purpose of diagnosis because the multicystic mass had grown bigger with time. If it was diagnosed malignant such as lung cancer by postoperative pathological examination, additional surgery needs to be planned for mediastinal lymph node dissection.

**Fig. 4:**
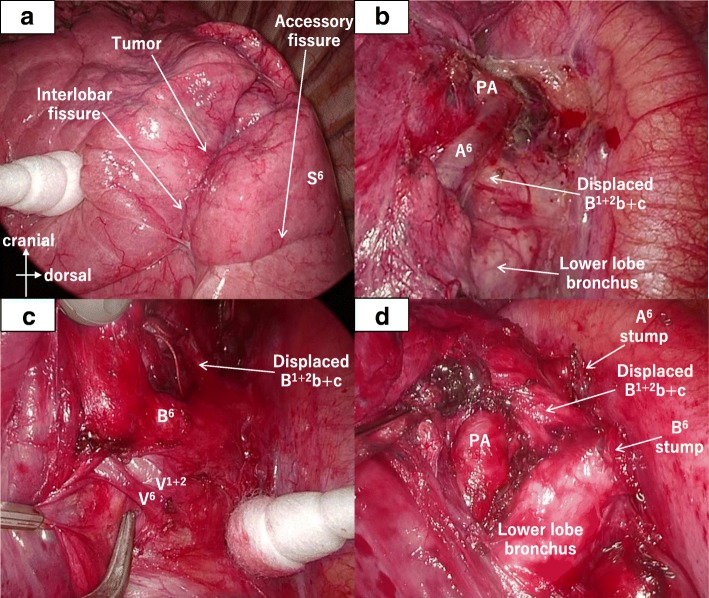
Intraoperative findings. There are incomplete accessory fissures between S^1+2^ and S^3^ and between S^6^ and the basal segment (**a**). The branch of A^6^ arises from the more proximal point than a normal anomaly and passes to the dorsal side of the displaced B^1+2^b+c (**b**). The branch of V^1+2^ passes between B^6^ and the bronchus to the basal segment and joins V^6^ at the dorsal side of the pulmonary hilum (**c**). The displaced B^1+2^b+c passes to the dorsal side of the main pulmonary artery (**d**)

Segmentectomy of S^1+2^b+c and S^6^ was performed by VATS with a 4 cm access thoracotomy at the fifth intercostal space of the anterior axillary line, a 1.5-cm access port at the sixth intercostal space of the posterior axillary line, and a 5-mm camera port at the seventh intercostal space of the middle axillary line. There were accessory fissures between S^1+2^ and S^3^ and between S^6^ and the basal segment that were largely fused. The intraoperative findings of the anatomy of the bronchi and pulmonary vessels were exactly the same as the preoperative CT findings (Fig. 4). At the cranial and dorsal sides of the pulmonary hilum, A^6^, which arose more proximal and passed to the dorsal side of the displaced B^1+2^b+c, was divided. Then, the displaced B^1+2^b+c was readily identified, and V^1+2^+V^6^, B^6^ and the displaced B^1+2^b+c were divided in sequence. After dividing the largely fused accessory fissure between S^6^ and the basal segment by stapler, A^1+2^c and A^1+2^b were divided. Finally, the largely fused accessory fissure between S^1+2^ and S^3^ was divided by stapler. The intersegmental line could be readily identified because of accessory fissures. If there was no accessory fissure, the technique that created a demarcation line between the inflated and deflated segment might be used. The operating time was 260 min, and the blood loss was minimal. The patient’s postoperative course was good. The pathological diagnosis was left lung abscess. The mass was a cyst connected to a bronchus. The wall structure was desquamated and replaced by the granulation tissue with inflammatory cells. Since there was no finding of a bronchial atresia in the resected specimen, the etiology of the lung abscess was considered as a bronchial cyst with recurrent infection.

## Discussion

The anatomical features of the present case were as follows: (1) left B^1+2^b+c branched from LMB and passed to the dorsal side of the left main PA, which was considered a displaced bronchus; (2) the branch of A^6^ arose from the more proximal point of the left main PA than in the normal anatomy and passed to the dorsal side of a displaced B^1+2^b+c; (3) V^1+2^ passed between B^6^ and the bronchus to the basal segment and joined V^6^ at the dorsal side of the pulmonary hilum; and (4) there were accessory fissures between S^1+2^ and S^3^ and between S^6^ and the basal segment. Because these anatomical features were identified on the preoperative 3D CT, segmentectomy of S^1+2^b+c and S^6^ by VATS was safely performed without misidentification of the anatomy.

**Fig. 5:**
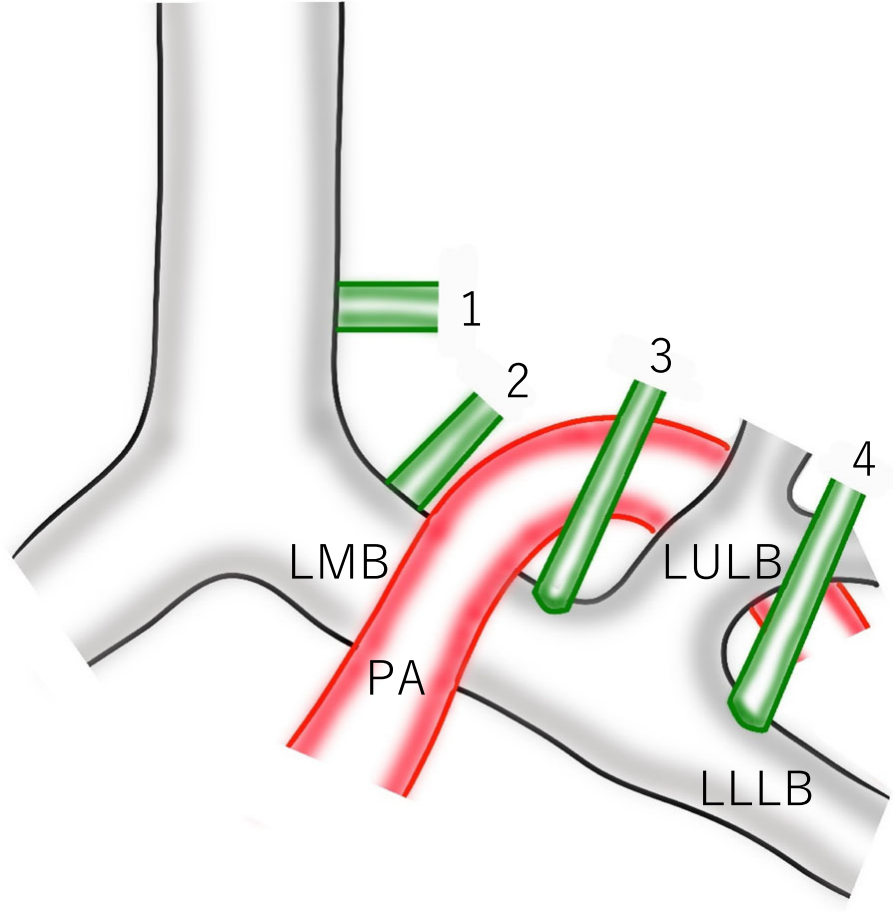
Schema of aberrant bronchi to the left upper lobe. Ghaye et al. classified them as follows: (1) eparterial (true left tracheal) bronchi; (2) eparterial (left “tracheal”) bronchi; (3) prehyparterial bronchi; and (4) posthyparterial bronchi. LMB, left main bronchus; PA, pulmonary artery; LULB, left upper lobe bronchus; LLLB, left lower lobe bronchus

With respect to aberrant bronchi to the left upper lobe, Ghaye et al. classified them as follows: (1) eparterial (true left tracheal) bronchi that arise from the trachea; (2) eparterial (left “tracheal”) bronchi that arise from the posterosuperior aspect of the LMB and pass to the dorsal side of the left main PA; (3) prehyparterial bronchi that arise from the anterosuperior aspect of the LMB and pass to the ventral side of the left main PA; and (4) posthyparterial bronchi that arise from the bronchus to the left lower lobe (Fig. 5) [9]. Before the development of CT, because the bronchus that passed to the dorsal side of the left main PA was identified by bronchography and intraoperative findings, it was called an eparterial bronchus [10, 11]. However, Oshiro et al. demonstrated that most of the origins of a displaced bronchus to the left upper division were lower than the inferior wall of the proximal PA on CT findings, and suggested that “eparterial bronchus” might not necessarily be the correct term [6]. While recent reports commonly used the term “a displaced bronchus,” “an eparterial bronchus” is an uncommon expression [7, 12]. Furthermore, there was another rare anatomical variation that the PA passed between the bronchus to the upper division and the lingual division with normal bronchial anatomy, which was reported by Melloni et al. as a variation of the course of the left PA [13]. Boyden et al. reported five cases with an eparterial bronchus, of which four cases had an eparterial bronchus arising from the left upper bronchus but not from the LMB [14]. These anomalies also suggested the possibility of variation of the course of the left PA rather than a displaced bronchus. With respect to a bronchus for the left upper division that passes to the dorsal side of the left main PA, displacement of the bronchus must be distinguished from a variation of the course of the left PA.

The branch of left A^6^ commonly arises from the interlobar part of the left PA between the origin of the lowermost artery of A^1+2^ and the origin of A^4+5^ (the common trunk of lingular segmental arteries) [15]. In the present case, the branch of A^6^ took its origin at the same level of the uppermost artery of A^1+2^ and A^3^ and passed to the dorsal side of a displaced B^1+2^b+c. Since the branch of A^6^ was identified preoperatively, it was able to be immediately found at the cranial and dorsal side of the pulmonary hilum during surgery and safely divided. This type of anomalous A^6^ can be injured or confusing if it is not identified preoperatively.

In the report about variations of the course of the PV by Shiina et al., the prevalence on the left side was 2.6%, which was less frequent than on the right side at 32.8% [16]. Among variations of the course of left V^1+2^, the variation that passed to the dorsal side of the pulmonary hilum and joined V^6^ or the inferior PV was more frequent than the others [17, 18]. In the present case, the left V^1+2^ passed between B^6^ and the bronchus to the basal segment and joined V^6^ at the dorsal side of the pulmonary hilum. Without preoperative identification on the 3D CT, there is a high potential of misidentifying an anomalous V^1+2^ as V^6^. In recent years, a preoperative 3D CT has contributed to a better understanding of the courses of pulmonary vessels and bronchi [19]. Because there are rare and unexpected variations of these structures, a preoperative 3D CT should probably be considered a routine examination as long as there is no contraindication to the use of contrast dye.

In the present case, there were accessory fissures between S^1+2^ and S^3^ and between S^6^ and the basal segment with incomplete lobulation. It has been reported that accessory fissures are incomplete in most cases [20]. Accessory fissures of the left upper lobe are common between the upper and lingular divisions, but rare between S^1+2^ and S^3^ [20]. Additionally, Oshiro et al. reported 10 cases with a displaced left upper division bronchus, of which 7 cases had an accessory fissure between the segment associated with a displaced bronchus and the remaining part of the left upper lobe [6]. An accessory fissure often coincides with an anatomical anomaly like a displaced bronchus [6]. In particular, if an accessory fissure is found between S^1+2^ and S^3^ during surgery, the presence of a displaced B^1+2^ that passes to the dorsal side of the left main PA must be suspected.

## Conclusions

VATS segmentectomy was performed for a patient with a displaced subsegmental bronchus and anomalous pulmonary vessels. A preoperative 3D CT may be helpful for the identification of anatomical anomalies. Furthermore, one should be alert to the fact that an anatomical anomaly might be present if an accessory fissure is found during surgery.

## Abbreviations

LMB: Left main bronchus
PA: Pulmonary artery
VATS,: Video-assisted thoracoscopic surgery
CT: Computed tomography
3D: Three-dimensional
PV: Pulmonary vein
